# Construct validation of a general movement competence assessment utilising active video gaming technology

**DOI:** 10.3389/fbioe.2023.1094469

**Published:** 2023-04-18

**Authors:** Jonathan Leo Ng, Chris Button

**Affiliations:** ^1^ Department of Health, Physical Education, and Sport, School of Education, College of Design and Social Context, RMIT University, Melbourne, VIC, Australia; ^2^ School of Physical Education, Sport, and Exercise Sciences, Division of Sciences, University of Otago, Dunedin, Otago, New Zealand

**Keywords:** dexterity, ecological dynamics, children, motion sensing, motor competence

## Abstract

**Introduction:** The assessment of children’s motor competence is an important concern as physical inactivity has been linked with poor movement quality and aspects of well-being such as low self-esteem. The General Movement Competence Assessment (GMCA) is a new instrument that was developed using active video gaming technology.

**Methods:** Confirmatory factor analysis was conducted to examine the internal validity of the GMCA in a sample of 253 typically developing children (135 boys and 118 girls), aged 7–12 years old (9.9 ± 1.6 years). Further, a second-order confirmatory factor analysis examined how the four constructs fit onto the higher-order variable of movement competence.

**Results:** Results revealed that the first-order four-construct model of the GMCA was a good fit (CFI 0.98; TLI 0.98; RMSEA 0.05). The second-order confirmatory factor analysis revealed that the four constructs loaded directly onto movement competence. It accounted for 95.44% of the variance which is approximately 20% more than the first-order model. The internal structure of the GMCA identified four constructs of movement competence (i.e., stability, object-control, locomotion and dexterity) based on the study sample.

**Discussion:** Performance trends in the general movement competence assessment support empirical evidence that movement competence improves as children age. Results suggest that active video games have considerable potential to help assess general motor competency in the wider population. Future work may consider the sensitivity of motion-sensing technologies in detecting developmental changes over time.

## Introduction

Compared to recent generations, 21st-century children have lower levels of movement competence (Hardy et al., 2013; Tester et al., 2014). We define movement competence as the capacity of an individual to adapt movements based upon affordances and action capabilities to produce goal-directed movement solutions that are effective and efficient. The functionality of emergent movement solutions arises from a group of inter-related constructs (such as balance, posture, and coordination). Evidence suggests that children that move competently are more likely to stay engaged in physical activity throughout their lives (Stodden et al., 2008; Lubans et al., 2010; Logan et al., 2014; Logan et al., 2015; Barnett et al., 2016). Yet it is concerning that there are increasing levels of sedentary behaviour and physical inactivity in children (Hallal et al., 2012; Farooq et al., 2018).

Video games have garnered much interest in the scientific community in the last decade as an alternative approach to getting children more physically active due to their mass appeal (LeBlanc et al., 2013). Active video games (AVGs) rely on motion-sensing technology to track the body movements of participants. These tracked movements are presented on a screen and embedded in progressions within the game. In particular, AVGs have been suggested as potential alternatives for monitoring and assessing movement competence (Giblin et al., 2014; Hulteen et al., 2015; Guess et al., 2017). Moreover, playing video games has been purported to solicit high levels of engagement, enjoyment and motivation (Hulteen et al., 2015; Lorenz et al., 2015; Smits-Engelsman et al., 2017).

A popular AVG system is the Microsoft Xbox 360 which utilises the Microsoft Kinect sensor (Microsoft, 2022) for motion detection. Studies involving the use of AVG systems such as the Microsoft Kinect have focused on its utility to monitor specific limb-joint movements (Choppin et al., 2014; Seo, Fathi, Hur, & Crocher, 2016; Napoli et al., 2017) and feasibility of its use as a rehabilitative tool (Camara Machado et al., 2017; Page et al., 2017). The Microsoft Kinect has been proven as a valid and reliable tool for use in tracking specific joint movements (Choppin et al., 2014; Guess et al., 2017; Napoli et al., 2017) as well as whole body movements (Hulteen et al., 2015; Guess et al., 2017).

### AVGs for assessing movement

The theoretical framework underpinning the development of the GMCA is ecological dynamics. The theory suggests that movement responses are a consequence of the dynamic interactions between individual, task and environmental constraints (Newell et al., 1991) that are scaled based on the affordances (or opportunities to act) and action capabilities of the individual (Dicks et al., 2009; Button et al., 2020). Thus, a wide variety of movement experiences and exploration in movement contribute to greater movement competence (Seifert et al., 2013; Rudd et al., 2020a). As a child ages, the compounding effect of movement experiences accumulates and progressively, the child becomes more competent in movement (Clark & Metcalfe, 2002). They become more sensitive to the affordances and are thus better able to adapt movement solutions based on their action-capabilities. In other words, movement competence is age-related and dependent on engagement in varied movement experiences.

Movement competence is typically assessed with movement assessment batteries (MABs) evaluating performance across three constructs; stability, locomotion and object-control (Gallahue & Ozmun, 2006; Barnett et al., 2016). The stability construct includes movements that emphasize balance in static and dynamic situations which require coordination of the trunk and axial limb movements. Specific examples can include balancing on one foot statically or dynamic forms such as walking along a line. The locomotion construct includes movements that allow body transportation from one point to another, such as sliding, leaping and galloping. The object-control construct describes manipulative, interceptive and prehension movement types such as catching, throwing or striking with and without additional equipment such as gloves or rackets and tools such as hammers. It typically involves the transmission of force to manipulate, move or receive an object.

MABs would often measure movement competence by evaluating combinations of or all of the three constructs. The origin of MABs have a clinical origin and they were purposefully designed to identify children with poor competence who would potentially require clinical intervention. These batteries however remain commonly used in the general population as a test of general movement competence in research and as an indication of performance in general programmes such as physical education and sports (Cools et al., 2009; Giblin et al., 2014; French et al., 2018). Importantly, the original intent for MABs to identify deviations from typically developing individuals lead to the design of simplified and isolated assessment tasks. This undoubtedly creates a ceiling effect when used with general populations. A ceiling effect is observed when a large portion of the sample attains high scores in a test resulting in a skewness in statistical analyses. It indicates that a test is not challenging enough for the specific cohort. Hence, in the context of MABs, the presence of ceiling effects in some MABs indicate a lesser sensitivity at identifying individuals who are performing at the higher end of the movement spectrum (French et al., 2018).

These MABs tend to involve the observation of discrete tasks that are often sport-related (such as balancing, running, jumping, throwing and striking) in tightly regulated settings (Cools et al., 2009; Ng & Button, 2018). The tasks common to these MABs are performed in isolation and often stripped of context to promote reliability (e.g., performing a static dribble as compared to dribbling in an actual physical activity and/or sport-orientated setting) (Pinder et al., 2011). The decontextualized design of these activities results in assessment tasks that are over-simplistic and prone to bias from cultural differences between countries (Cools et al., 2009). This can result in unfair bias against children that may have not had the same opportunities to participate in sports and certain physical activities that involve the use of the assessed movement skill (Rudd et al., 2020b; Smits-Engelman et al., 2022). Essentially, these MABs are being used for purposes that they were not originally designed for.

Process and product-based assessments primarily differ in assessment forms. Process-based assessments utilise observational criteria to determine the quality of a particular movement (e.g., running form) that are criterion-referenced. Product-based assessments measure quantitative, outcome variables (e.g., running duration) and are often referenced with normative samples. Many MABs adopt criterion-based reference in evaluating movement competence by comparing the performances of individuals based on the ‘correctness’ of the technique employed in specific tasks (e.g., how to throw or catch a ball). In order to reliably reproduce the assessment settings, MABs are composed of static and isolated tasks that downplay or remove typical affordances that are present in a naturalistic setting (e.g., throwing a pass to an unmarked team-mate in a game situation). The technique-focused outcome typically requires a specific set of desired movement solutions (Kane, 2013). Hence, individuals who may be successful in achieving outcome goals through alternative, innovative forms of movement are penalised. Arguably, the assessment tasks do not allow children to demonstrate their ability to react and respond to changing constraints that are typical in an authentic setting (Newell et al., 1991; Cools et al., 2009).

In recent times, some contemporary MABs have begun adopting a more purposeful approach in the design of assessment tasks as a consequence of growing concerns about their ability to distinguish between more or less competent individuals. These validated MABs include more dynamic tasks such as obstacle courses and game-based formats to evaluate movement in more representative contexts (e.g., Tylor et al., 2018; Flôres et al., 2021; Morley et al., 2021).

In our opinion, the game-based virtual environment of AVGs has considerable potential in offering a dynamic environment within controlled settings to potentially evaluate movement competence. Physical interaction with AVGs allows individuals to demonstrate their ability to accurately scale movement responses based on the changing affordances presented in modifiable tasks. Notably, the use of motion-sensing video game technology would ease some logistical constraints of space and equipment (e.g., typical requirements of MABs are large, unobstructed rooms such as school gyms) as well as the need for specialised training and assessors required by many MABs (Cools et al., 2009; Giblin et al., 2014). As gaming and e-sports continue to grow in popularity around the world (Franks et al., 2022), an active gaming platform may offer an inclusive means to develop general movement competency assessments that are less sensitive to cultural differences.

A new movement assessment instrument, the General Movement Competence Assessment (GMCA) was developed using the technology of the Microsoft Kinect with a series of AVGs created to assess various attributes of movement. In a previous study, Ng and others (2020) proposed the inclusion of a new movement construct (i.e., dexterity) in addition to the three commonly accepted constructs of movement competence (i.e., stability, locomotion and object-control) to provide a more encompassing description of movement competence suitable for the general population of healthy children. Before this work, dexterity had not been previously identified as an interdependent construct in the model of movement competence. Although the exploratory factor analysis showed that over 70% of the variance in performance on the GMCA games was explained by a four-construct model, a cautious approach was taken in generalising results with a limited sample size of 83 children (aged 8–10 years). This prompted further validation work with a focus on increasing sample size across wider age groups.

Robust assessments of children’s movement competency are of fundamental importance given the potential impact on health given declining levels of physical activity globally. Unfortunately, many existing movement assessment tools that were designed to classify children with very low competency over-emphasise technique instead of adaptation to constraints as well as creative movement alternatives to overcome movement problems. We propose that AVGs offer a promising platform to develop a new movement competency assessment tool that is suitable for use in the general population of children. The primary aim of the present study was to assess the internal structure of the GMCA in a sample of children 7–12 years old through confirmatory factor analysis of the proposed four-construct model consisting of dexterity, stability, locomotion and object-control (Ng et al., 2020).

## Materials and methods

### Participants

The study sample consisted of 259 typically developing children, ranging from 7–12 years of age (*M* age = 9.97 years, *SD* = 1.61) from a variety of ethnicities. There were 138 boys (*M* age = 10.12 years, *SD* = 1.57) and 121 girls (*M* age = 9.80 years, *SD* = 1.64). All children were recruited through a convenience sampling method from a public primary school in Dunedin, New Zealand. As the GMCA had not been programmed to accommodate individual differences for children with disabilities, children with any physical impairment or disabilities (e.g., visual, hearing impaired, children with cerebral palsy, etc.) were excluded from the study. Written informed consent was attained from both parents and child participants and approval for the study (17/071) was obtained from the Human Ethics Committee of the participating institution.

Simulation work suggest that sample sizes for confirmatory factor analysis with maximum likelihood varies. Jak et al. (2014) classify sample sizes of *n* ≤ 250 as “small”. However, power estimates conducted for this study suggested sample sizes of between 200–300 for the test of closeness of fit (PCLOSE) for values of 0.769 and 0.928, respectively (MacCallum et al., 1996). Additionally, considering the degrees of freedom (*df*) for the extracted four-construct model of Ng et al. (2020) the sample size (*n* = 259) for the study was suitable based on the recommendations of MacCallum and others (1996) and fits the recommendations of other simulation work (Marsh et al., 1988; Kim, 2005; Wolf et al., 2013).

### The GMCA application

The GMCA is a custom-written application (programmed in C++) that utilises the open-sourced Kinect for Windows Software Development Kit 2.0 to work with the Microsoft Kinect 2.0 system (Microsoft, Redmon, WA). The Kinect system consists of an infrared emitter, colour video and depth sensor. It tracks movements from the reflection of emitted infrared rays. The video and depth sensor captures three-dimensional movements and automatically locates and detects 25 joint centres of the human body.

The GMCA consists of five custom-programmed active video ‘games’. The five games are called *Balance, Precision, Control, Swiftness*, and *Interception*, with each game increasing in task difficulty as one meets the movement demands required by each stage (see Supplementary Table S1). The order of the various GMCA games were presented as follows^1^: 1) Precision_unimanual, 2) Balance, 3) Precision_symmetrical, 4) Control, 5) Swiftness, 6) Precision_asymmetrical and 7) Interception. Once started the games would run automatically without the need for external administrators. Performance scores for each task were calculated based on how well children completed (or not) the progressively difficult levels of each game. Thus, if an individual did not do well in earlier stages of the game it was expected that they would not be as accomplished in the later stages.

The spatiotemporal demands of each task were pre-programmed into the software and these were modified to increase once a participant reached a specified threshold of achievement. Thus, the relative demands placed on the individual in terms of difficulty and complexity increased at each stage (e.g., the game *Precision* started with symmetrical pathways to asymmetrical pathways resulting in an increased difficulty and complexity of the task). Hence, individuals had to adapt their movement responses appropriately and it was expected that individuals at the higher end of the movement spectrum (Ng & Button, 2018) would have better performances even in the more complex stages of the games.

### Measured variables of GMCA games

The GMCA assesses movement competence based on the measured variables from each game (for details see: Ng et al., 2020). For example, in the *Balance* game, a static balance pose is considered successful when the prescribed hands and feet positions were held for 3 s. The measured variable for *Balance* was the total number of successful poses held for each of the three stages. Importantly, the more competent individual would be better able to adapt the emergent postural control strategies and body configurations to suit the demands of the task. Recorded variables for the game, *Precision*, included the total time taken to move an object around set courses for three levels of difficulty. Measured variables from *Control* included the total number of balls used for the game and the total number of balloons popped. *Control* required individuals to first, juggle a virtual ball on the screen, then manoeuvre the ball to ‘pop’ a balloon that appeared at random locations on the screen. Hence, a proficient player would be able to control the juggle of a ball and use that same ball to pop the balloons that appeared throughout the game. On the other hand, a less able player would use more balls since a ball was ‘replaced’ when it was ‘lost’ (i.e., juggled out of control). Variables from the *Swiftness* game included the total amount of time taken to move between set places in the play area for each of the two levels of difficulty. Finally, the game, *Interception*, required participants to primarily, ‘save’ spaceships by hovering a hand over them as they appeared at randomised locations on the screen at the same time. A secondary task required participants to ‘intercept’ stray asteroids by touching them using the other free hand. The asteroids were programmed to take random flight paths and fly at random speeds throughout the game. The measured variables for *Interception* were the number of spaceships ‘saved’ and the number of spaceships ‘lost’ (or destroyed by the stray asteroids).

### Equipment and test layout

The standing height and weight of participants were measured with a portable stadiometer and a digital scale (UC-321, A&D Company Limited). The GMCA games were displayed on a Sony KDL-40EX400 40-inch 1,080 pixels HDTV. The TV was set upon a standing console 0.8 m from the ground. The Kinect Sensor was placed directly in front of the TV facing the game area. All trials were recorded to serve as a reference in the event of discrepancies.

In the present study, the game area measured 2.05 by 2.55 m. The distance between the Kinect sensor and the game area measured 2 m away from the front edge of the Kinect sensor to the front boundary of the play area. Play area boundaries were marked out with high-contrast coloured tape on the ground.

### Procedure

Participants’ anthropometric measures were recorded 1 week before data collection. Age was calculated by subtracting the date of the testing date from the birth date of each child. The start and end of the GMCA application were controlled by an experimenter operating the computer at each testing station. The GMCA does not require any specialised training, nor does it require the presence of a tester with specialised knowledge of motor performance for the GMCA was programmed to run automatically. All participants engaged in the GMCA trial once immediately after their familiarisation trial. Including familiarisation, the entire GMCA test duration ranged from approximately 10–18 min per individual. More competent individuals completed GMCA trials faster^2^.

### Data analysis

#### Internal consistency

Data were analysed using the Statistical Package for the Social Science (SPSS; version 24, IBM Corp., Armonk, NY) with statistical significance set at *p* ≤ 0.05. To determine the degree of homogeneity of measured variables from each movement construct of the GMCA, internal consistency reliability was analysed by omega coefficient; *ω* (McDonald, 1999). Reliability was accepted at *ω* > 0.7 (Nunnally & Bernstein, 1994).

### Confirmatory factor analysis

A proposed four-construct model of the GMCA was validated for the study sample using confirmatory factor analysis. To determine the suitability of the data for factorial analysis, data were screened using Kaiser-Meyer-Olkin (KMO) and Bartlett’s test of sphericity. Data is suitable for factor analysis when the KMO value is more than 0.6 (Kaiser & Rice, 1974) and when Bartlett’s test is significant (*p* < 0.05). When assumptions were met, confirmatory factor analysis was conducted using the maximum likelihood method of estimation in AMOS 24.

The fit of the tested model was interpreted from various fit indices. On top of the chi-square (χ2) statistic and df, other goodness-of-fit indices were used to determine model fit. These were the χ2 divided by the df (χ2/df), Comparative Fit Index (CFI; Bentler, 1990), Lewis-Tucker Index (TLI; Tucker & Lewis, 1973), root mean square of approximation (RMSEA; Steiger & Lind, 1980; Browne & Cudeck, 1993) with confidence intervals (CI) and probability of the test of close fit (PCLOSE; Hu & Bentler, 1999).

The *χ* ^2^ statistic measures the overall fit of the model with a higher probability (*p* > 0.05) indicating a closer fit between the tested model and the perfect fit (Bollen, 1989). Instances of good fitting models being rejected with the test of exact fit due to the large *χ* ^2^ statistic relative to the *df* have been highlighted in the literature (Jöreskog & Sörbom, 1993). Thus, other alternative indices of fit were used to address the limitations associated with the *χ* ^2^ statistic.

Alternate fit indices (i.e., *χ* ^2^/*df*, CFI, TLI and RMSEA) are typically used as adjuncts to the *χ* ^2^ statistic. χ^2^/*df* provides an indicator of fit with values of less than 2 being considered an adequate fit (Wheaton et al., 1977). The CFI is a revision of the Normed Fit Index (NFI; Bentler & Bonnet, 1980) that takes sample size into account since a limitation of NFI is the underestimation of fit in small samples (Byrne, 2013). The TLI yields values from 0.0 to 1.0. Values closer to 1.0 are indicative of a good fit. CFI and TLI values more than 0.9 were interpreted as “acceptable”, while values more than 0.95 were “good” (Hu & Bentler, 1999).

The next fit statistic, RMSEA, is postulated to be one of the most informative fit indexes as it considers the error of approximation through the provision of CIs (Browne & Cudeck, 1993). RMSEA values of less than 0.05 are indicative of a “good” fit; 0.05 to 0.08, “fair” and 0.08 to 0.10, “mediocre” (Browne & Cudeck, 1993). Non-etheless, Hu and Bentler (1999) propose that RMSEA values of up to 0.06 can still be considered a good fit. CI substantiates the RMSEA value by providing additional information regarding the precision of estimates (MacCallum et al., 1996). For example, if the lower bound of the RMSEA’s CI is above 0 and less than 0.05, then the probability of the *χ* ^2^ statistic being less than 0.05 is expected (MacCallum et al., 1996). Additionally, if the upper bound of the CI is above 0.05, it would be an indication of a plausible good-fitting model.

PCLOSE is a test for the closeness of fit. Specifically, it tests the hypothesis that the RMSEA value is “good” for the sample population. The probability for the PCLOSE test should be *p* > 0.50 (Browne & Cudeck, 1993).

The four-construct model of the GMCA was validated in this population of children aged 7–12 years old. Modification indices generated by AMOS 24 were only considered if proposed modifications were theoretically grounded, else, modifications made would reflect minute changes in the model according to the nuances of the sample (Byrne, 2013). In addition, a second-order confirmatory factor analysis was conducted to examine if the four constructs loaded onto the higher-order variable of movement competence.

## Results

### Internal consistency

Preliminary examination confirmed there was no significant difference in age within the sample for boys and girls (*p* = 0.11). Table 1 shows the results of the internal consistency reliability analysis for each of the four constructs measured by the GMCA. For all constructs, omega coefficients were above the recommended 0.7 value.

**TABLE 1:** Internal consistency reliability of the four GMCA constructs.

Construct	Variables included	Omega coefficient
Stability	Balance stage 1	0.74
	Balance stage 2	
	Balance stage 3	
Dexterity	Spaceships stage	0.96
	Spaceships lost	
Locomotion	Swiftness stage 1	0.87
	Swiftness stage 2	
Object-control	Balls used	0.87
	Precision stage 1	
	Precision stage 2	
	Precision stage 3	

### Confirmatory factor analysis

Confirmatory factor analysis with maximum likelihood estimation was conducted to test the internal structure of the GMCA as extracted from a previous study (Ng et al., 2020). Assumptions testing indicated that the sample data was suitable for factorial analysis. The KMO value was 0.86 which indicated excellent suitability and Bartlett’s test was significant (*p* < 0.001).

**FIGURE 1:**
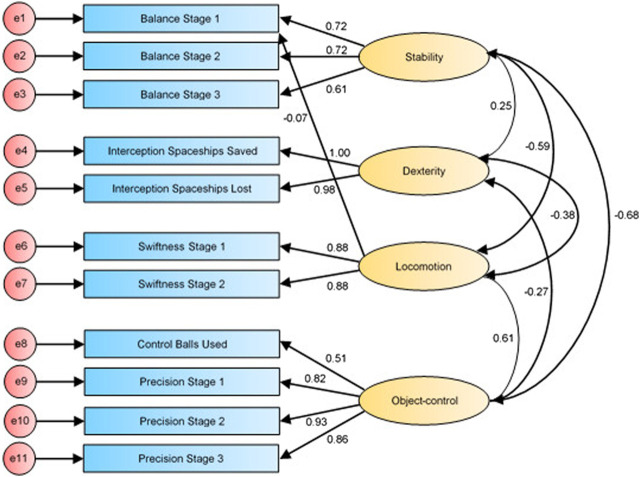
First-order factor structure of the GMCA (initial fit; Model 1).

**FIGURE 2:**
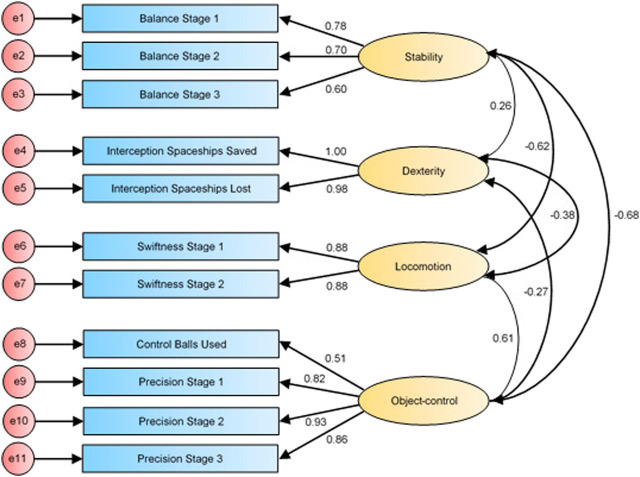
First-order factor structure of the GMCA (re-specified fit; Model 2).

**FIGURE 3:**
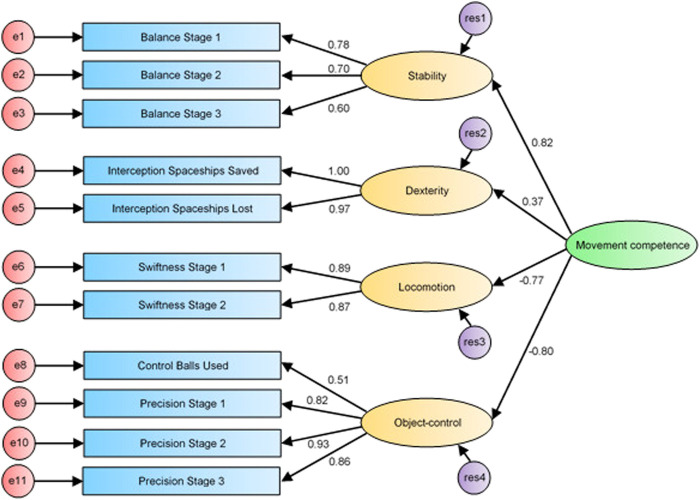
Second order CFA model for movement competence.

The path diagrams (i.e., Figures 1–3) of confirmatory factor analysis comprise all 11 measured variables included in the analysis as well as the four specified constructs of the GMCA. Each construct consists of the measured GMCA game variables (also known as observed variables) and is influenced by a random measurement error, indicated by the associated error term (e.g., e1, e2, e3, etc.). Each observed variable regresses onto its respective construct. Finally, the constructs co-vary *via* the corresponding covariate arrows in path diagrams from the specified model.

### First-order factor analysis for the four-construct model of the GMCA

The specified model (Model 1; see Figure 1) was based on the four-construct model extracted from the exploratory factor analysis of Ng and others (2020). The four constructs measured were balance, locomotion, object-control and dexterity. The variable, Balance Stage 1, was specified to double load onto the locomotion construct as dynamic balances are proposed to influence the performance of locomotion tasks (Bril & Brenière, 1993).

The initial confirmatory factor analysis for the specified four-construct model (Model 1; see Figure 4.2) found an adequate fit (*χ* ^2^(37) = 60.006; *p* = 0.010; *χ* ^2^/*df* = 1.622; CFI = 0.988; TLI = 0.983; RMSEA = 0.049, CI 0.024–0.071; PCLOSE = 0.500).

In model 1, the standardized regression weight of Balance Stage 1 loading onto the locomotion construct was −0.072. Notably, factor loadings (i.e., regression weight) of less than 0.4 are not considered valuable to the overall model fit (Sireci, 2007). Thus, the initial model (Model 1; see Figure 1) was re-specified with the removal of the double loading between Balance Stage 1 and the locomotion construct and confirmatory factor analysis was conducted again.

The second model (Model 2; see Figure 2) supports the data characteristics well based on fit indices (*χ* ^2^(38) = 60.588; *p* = 0.011; *χ* ^2^/*df* = 1.594; CFI = 0.989; TLI = 0.983; RMSEA = 0.048, CI 0.023–0.070; PCLOSE = 0.533) which accounted for 74.85% of the variance. Modification indices suggested several ways to enhance the model fit, however, the suggested changes were not theoretically justifiable.

### Second-order factor analysis for movement competence

With the first-order confirmatory factor analysis establishing a good fit of the specified four-construct model of the GMCA, a second-order confirmatory factor analysis was undertaken that specified for each construct to load onto the higher-order variable of movement competence. The four second-order latent variables of stability, dexterity, locomotion and object-control were specified to load directly into movement competence (see Figure 3).

Results indicated that an adequate fit was achieved (*χ* ^2^(40) = 67.376; *p* = 0.004; *χ* ^2^/*df* = 1.684; CFI = 0.986; TLI = 0.981; RMSEA = 0.052, CI 0.029–0.072; PCLOSE = 0.429) and accounted for 95.44% of the variance (approximately 20% more variance than the first order model accounted for). Table 2 provides a tabled comparison of the various fit indices for the three tested models.

**TABLE 2:** Fit indices of each specified model.

Model	Description	*χ* ^2^	*df*	*p*	*χ* ^2^/*df*	CFI	TLI	RMSEA [CI]	PCLOSE
1	Hypothesized four-construct model	60.01	37	0.01	1.62	0.99	.98	0.05 [0.02, 0.07]	0.50
2	Four-construct model without double loading	60.59	38	0.01	1.59	0.99	.98	0.05 [0.02, 0.07]	0.53
3	Movement competence model	67.38	40	0.00	1.68	0.99	.98	0.05 [0.03, 0.07]	0.43
*Abbreviations: χ* ^2^, chi-square; *df*, degrees of freedom; *p*, probability; CFI, comparative fit index; TLI, Lewis-Tucker index; root mean square of approximation, RMSEA; CI, confidence interval; PCLOSE, probability of the test of close fit.									

## Discussion

The present study examined the internal structure of the GMCA. Both the first and second-order confirmatory factor analysis models indicated a good fit, particularly to the sample data. The specified internal structure of the GMCA, existing as a four-construct model was empirically supported. These results confirm that the GMCA is a multi-dimensional assessment and that all four constructs have varying degrees of influence on the description of movement competence. The findings supplement previous studies that have highlighted the interdependence between movement constructs (Bril & Brenière, 1993; Davids et al., 2000; Rudd et al., 2016). Consequently, all constructs that defined it should ideally be considered when evaluating the general movement competence of typically developing individuals. This may suggest the need to supplement MABs that measure three specific movement constructs (e.g., locomotion, object-control and stability) with other forms of assessment to gain a better description of general movement competence, especially in typically developing populations.

Importantly, the model confirmed that another movement construct (i.e., dexterity) is required to differentiate the movement competence of children of different ages. Results of confirmatory factor analysis for the GMCA game variables loading onto the construct of dexterity provide a working definition for dexterity as the act of using/moving both sides of the body independently, in order words, the ability to be competent bilaterally (including bimanual coordination). Indeed, some MABs include dexterous tasks that require successful coordination of both sides of the body to achieve outcome goals (e.g., Bruininks-Oseretsky Test of Motor Proficiency second edition (BOTMP-2); Bruininks, 2005). However, dexterity has not yet been identified as an independent construct of movement competence nor included as an independent construct in other validated MABs. For example, results for the dextrous tasks loaded onto the construct defined as gross motor skill construct in BOTMP-2 (Bruininks, 2005).

With our definition of movement competence, the inclusion of dexterity as an additional construct in the model of movement competence may provide supplementary evidence for the traits of competent individuals and better distinguish between individuals residing across the movement competence spectrum. Additionally, evaluating dexterity can potentially supplement MABs that examine other commonly accepted constructs of movement (e.g., stability, locomotion and object-control; Gallahue & Ozmun, 2006). This would also respond to previous recommendations calling for MABs to be supplementary to each other (Cools et al., 2009; Rudd et al., 2015).

For the version of the GMCA used in this study, the Balance game was updated to only include one-leg balances as compared to a mix of two- and one-leg balances for an earlier version of GMCA (Ng et al., 2020). In the extracted model from Ng and others (2020), the one-leg balance variable is loaded onto the locomotion construct. Hence, an exact replica of the model would mean that all the Balance variables (from the updated version of GMCA) would need to be specified to co-vary with the locomotion construct. Had this been done, it would be akin to specifying that the stability and locomotion construct co-vary which confirmatory factor analysis procedures by default requires (i.e., all constructs of specified models must co-vary). Thus, as the model (see Figure 2) was already specified to co-vary between the stability and locomotion construct, the double loading of Balance Stage 1 to the locomotion construct was redundant (Brown, 2014). In model 2 (see Figure 2), the double loading of Balance Stage 1 was removed which resulted in a marginally better fitting model. Large improvements to the model after re-specification was never expected since the double loading only had a negligible standardised regression weight of −0.072. Hence, a slight improvement in the model fit was expected with this modification.

The second-order confirmatory factor analysis model (see Figure 3) revealed that the construct with the strongest correlation with movement competence was stability (*r* = 0.82). This strong association was closely followed by the object-control (*r* = −0.80), and locomotion (*r* = −0.77) constructs, and then by dexterity (*r* = 0.37). Although the dexterity construct was found to load the weakest (*r* = 0.37) onto movement competence, it still makes an important contribution to the overall model fit. The dexterity construct was made up of variables from the Interception game which measured the ability of individuals to use both sides of their body independently to achieve outcome goals. Findings from Rudd and others (2016) indicate the coordination construct was made up of assessment tasks from the Körperkoordination Test für Kinder (Kiphard & Schilling, 2007) that focuses on bilateral coordination competence. Hence, despite the weak loading, it is suggested that the role of dexterity should not be neglected for its role in describing general movement competence (Bernstein, 1996).

Individuals at the higher end of the spectrum may be more proficient at dexterous tasks based on their varied movement experiences in relation to the movement dynamics (Bernstein, 1996; Logan et al., 2014; 2015; Morley et al., 2021). From an ecological dynamics perspective, engaging in an enriched environment provides for varied movement experiences (Ng and Button, 2018; Scheuer et al., 2019; Button et al., 2020). These remain critical for individuals in all life stages since the varied movement experiences would increase an individual’s sensitivity to their action capabilities (Clark & Metcalfe, 2002; Hulteen et al., 2015). An individual’s increased awareness of action capabilities also decreases the risks for potential injury since the varied movement experiences contribute to an increased sensitivity to the affordances or “opportunities to move”. Importantly, varied movement experiences allow the emergence of dexterous movements to be developed and refined (Bernstein, 1996) which may suggest that individuals who have had a wider range of movement experiences may also reside at the higher end of the movement spectrum. Thus, the finding of dexterity as a construct of movement competence is significant.

Lastly, the results of the present study indicate that stability remains the most influential construct of movement competence. Compared to the other constructs, stability explained the largest percentage of variance (20.5%) from the exploratory factor analysis conducted by Ng and others (2020). Notably, previous studies highlighting the importance of stability competence on other movement constructs have advocated strongly for its inclusion in movement competence assessments (Davids et al., 2003; Luz et al., 2016; Rudd et al., 2016; Anderson, Button, & Lamb, 2022).

### Assessment form

To measure movement competence, process-based assessment approaches have been recommended (Ulrich, 2000; Stodden et al., 2009). Indeed, the GMCA utilises a product-based approach towards the measurement of movement competence. Process and product-based assessments primarily differ in assessment forms. Although the validity of both forms of assessments to measure movement skills have been raised before (Stodden et al., 2008; Logan et al., 2012), previous studies have suggested associations between the two (Roberton & Konczak, 2001; Miller et al., 2007; Mally et al., 2011), thus, highlighting that both forms have their merits and that results from both assessment forms are valid for the purposes that they were designed for.

From an ecological dynamics perspective, the implications on practice are that learning or assessment tasks in the movement context should in design and execution strive to ensure that the link between perception and action remains and is not left decoupled by design. When assessment tasks are decoupled or decontextualized, it limits the opportunity to provide an accurate description of movement competence. A simulated assessment environment should have elements of the performance environment to ensure representativeness (Chow et al., 2007; Dicks et al., 2009; Pinder et al., 2011). This allows individuals to demonstrate their capacity to adapt efficient movement forms that are self-organised based on inherent individual differences (Schöllhorn et al., 2002) in addition to the demands presented by dynamic movement situations which are found in activities of daily living physical activity and sport at all levels of participation. The effectiveness, efficiency and quality of movement can then be judged based on a contextualised movement problem that keeps the perception-action coupling intact.

Understanding the process of movement or assessing its quality is suggested to be an important feature in determining the efficiency of movement (Ulrich, 2000) and process-based MABs often assess children’s movement skills based upon a mature, expert-like form (Stodden et al., 2008). Notably, that approach fails to consider the influence of individual differences on movement responses (Vella et al., 2023). Importantly, variability of and within observed movements is inherently present due to the unique individual differences of every individual (Chow et al., 2007). In addition, there are no universally ideal or expert-like patterns of movement (Davids et al., 2013; Seifert et al., 2013). Hence, the GMCA was developed as a product-based assessment that is concerned with the movement outcome since the process of executing movement would be unique to each individual, based upon their action capabilities and interaction with task and environmental constraints.

### Strengths, limitations and future directions

One of the merits of this study is its utilisation of relatively low-cost, portable video game technology that can be operated without specialised training to help ease some of the constraints of current MABs such as the need for trained assessors (Cools et al., 2009). As the GMCA was written with an open-source application, there is potential to further programme customised games that could suit various population samples. There is potential for the GMCA to also be used as a supplementary teaching aid for the general population. There is also considerable promise for the GMCA with its AVG modality to be used in conjunction with intervention programmes for clinical populations (Camara Machado et al., 2017; Page et al., 2017). The large sample of children utilised and naturalistic settings (i.e., in school classes) were also strengths of the study. Notably, our results suggest stability competence has a critical influence on the other three movement constructs which signals future work to establish if the influence is variable across the age groups.

There are some limitations in this study. First, the model fit was specific to the study sample and some caution has to be heeded in generalising to other populations. Future studies should consider its validity in other populations. Assessing validity is an ongoing process of evaluating data that is first derived from a specific population. Hence, more than one source of evidence is necessary (Messick, 1989). Furthermore, as evident in past validation studies of MABs, a critical limitation has been raised concerning the incongruent results found when particular MABs were used in different populations from normative samples (Chow et al., 2006; van Waelvelde et al., 2008; Bardid et al., 2016). Hence, future studies should consider validating the GMCA in other populations to further supplement the validity evidence (Zumbo & Chan, 2014). Secondly, the efficacy of any assessment will be in its discriminative validity to detect changes over time however this was not yet established in this study. Therefore, to further validate the GMCA, future research should determine the GMCA’s sensitivity in tracking developmental changes. Lastly, it is also recommended that the relationship between dexterity and overall movement competence be explored further to inform the design of future movement assessments that are suitable for use amongst the general population. Future studies should explore the role of dexterity in distinguishing children across the spectrum of movement competence. This could be achieved through concurrent validation studies between the dexterous tasks of the GMCA and the other validated assessment batteries such as the Brunicks-Oseretsky Test of Motor Proficiency (Bruininks, 2005) and Movement ABC-2 (Henderson et al., 2007).

## Conclusion

The validity evidence obtained from this large sample of school children confirms the GMCA measures general movement competence *via* a four-construct model. Importantly, this study reaffirms that dexterity can be considered an independent construct in the model of movement competence (Ng et al., 2020).

The GMCA does not require specialised training, it is relatively easy to use, and it can be adapted for use with other motion-sensing technologies^3^ (e.g., Azure Kinect DK, Microsoft, 2023) since C++ is a flexible and adaptable language. In this study, the GMCA utilised the technology of video games to provide interactive dynamic movement assessment tasks. The use of dynamic over static tasks in the GMCA maintains the perception-action coupling which is more representative of how we interact in the real world through movement. Additionally, dynamic tasks allow individuals to demonstrate their ability to adapt and respond to changing task constraints. As a product-based measure, it affords multiple movement solutions to be used instead of focusing on one ‘ideal’ solution which may not be as inclusive for all due to our unique individual differences and movement preference.

Indeed, the potential to incorporate the use of motion-sensing technology as a novel supplement can complement current methods of assessing movement competence and may prove useful to practitioners in the industry.

## Data Availability

The original contributions presented in the study are included in the article/Supplementary Material, further inquiries can be directed to the corresponding author.

## Footnotes

1 The order of GMCA games could be manually chosen if needed. The default sequence of the GMCA broke up the various stages of the Precision game in order to prevent fatigue in the arms. These broken up stages were ordered progressively with increasing difficulty.

2 More competent individuals recorded faster completion time for GMCA games such as Precision and Swiftness were time based. Hence, more competent individual completed those games faster, thus, resulting in faster overall completion times.

3 Microsoft has discontinued manufacturing the Kinect 2.0. However, with C++ as the programming language for the GMCA, the opportunity remains for the application that is also open source to be adapted for use with other commercially available motion-sensing devices.

## References

[B1] Anderson N. Button C. Lamb P. (2022). The effect of educational gymnastics on postural control of young children. Front. Psychol. 13, 936680. 10.3389/fpsyg.2022.936680 36033080 PMC9399810

[B2] Bardid F. Huyben F. Lenoir M. Seghers J. De Martelaer K. Goodway J. D. (2016). Assessing fundamental motor skills in Belgian children aged 3–8 years highlights differences to US reference sample. Acta Paediatr. 105 (6), 281–290. 10.1111/apa.13380 26933944

[B3] Barnett L. M. Lai S. K. Veldman S. L. Hardy L. L. Cliff D. P. Morgan P. J. (2016). Correlates of gross motor competence in children and adolescents: A systematic review and meta-analysis. Sports Med. 46 (11), 1663–1688. 10.1007/s40279-016-0495-z 26894274 PMC5055571

[B4] Bentler P. M. Bonett D. G. (1980). Significance tests and goodness of fit in the analysis of covariance structures. Psychol. Bull. 88 (3), 588–606. 10.1037/0033-2909.88.3.588

[B5] Bentler P. M. (1990). Comparative fit indexes in structural models. Psychol. Bull. 107 (2), 238–246. 10.1037/0033-2909.107.2.238 2320703

[B6] Bernstein N. A. (1996). “On dexterity and its development,” in Dexterity and its development. Editors Latash M. Turvey M. T. (Mahwah, NJ: Lawrence Erlbaum), 3–244.

[B7] Bollen K. A. (1989). Structural equations with latent variables. New York, NY: Wiley.

[B8] Bril B. Brenière Y. (1993). “Posture and independent locomotion in early childhood: Learning to walk or learning dynamic postural control?,” in The development of coordination in infancy. Editor Savelsbergh G. J. P. (Amsterdam: Elsevier Science Publishers), 97, 337–358.

[B9] Brown T. A. (2014). Confirmatory factor analysis for applied research. New York, NY: Guilford Publications.

[B10] Browne M. W. Cudeck R. (1993). “Alternative ways of assessing model fit,” in Testing structural equation models. Editors Bollen K. A. Long J. S. (Newbury Park, CA: Sage Publications), 136–162.

[B11] Bruininks R. H. (2005). Bruininks-oseretsky test of motor proficiency. 2nd ed. Minnesota, MN: AGS Publishing Circle Pines.

[B12] Button C. Seifert L. Chow J. Y. Davids K. Araujo D. (2020). Dynamics of skill acquisition: An ecological dynamics approach. Canada: Human Kinetics Publishers.

[B13] Byrne B. M. (2013). Structural equation modeling with AMOS Basic concepts, applications, and programming. Second ed. London: Taylor & Francis.

[B14] Camara Machado F. R. Antunes P. P. Souza J. D. M. Santos A. C. D. Levandowski D. C. Oliveira A. A. D. (2017). Motor improvement using motion sensing game devices for cerebral palsy rehabilitation. J. Mot. Behav. 49 (3), 273–280. 10.1080/00222895.2016.1191422 27593342

[B15] Choppin S. Lane B. Wheat J. (2014). The accuracy of the Microsoft Kinect in joint angle measurement. Sports Technol. 7 (1-2), 98–105. 10.1080/19346182.2014.968165

[B16] Chow J. Y. Davids K. Button C. Koh M. (2007). Variation in coordination of a discrete multiarticular action as a function of skill level. J. Mot. Behav. 39 (6), 463–479. 10.3200/jmbr.39.6.463-480 18055353

[B17] Chow S. M. Hsu Y. Henderson S. E. Barnett A. L. Lo S. K. (2006). The movement abc: A cross-cultural comparison of preschool children from Hong Kong, taiwan, and the USA. Adapt. Phys. Act. Q. 23 (1), 31–48. 10.1123/apaq.23.1.31

[B18] Clark J. E. Metcalfe J. S. (2002). “The mountain of motor development: A metaphor,” in Motor development: Research and reviews. Editors Clark J. E. Humphrey J. (Reston, VA: NASPE Publications), 2, 163–190.

[B19] Cools W. De Martelaer K. Samaey C. Andries C. (2009). Movement skill assessment of typically developing preschool children: A review of seven movement skill assessment tools. J. Sports Sci. Med. 8 (2), 154–168. 24149522 PMC3761481

[B20] Davids K. Araújo D. Vilar L. Renshaw I. Pinder R. (2013). An ecological dynamics approach to skill acquisition: Implications for development of talent in sport. Talent Dev. Excell. 5 (1), 21–34.

[B21] Davids K. Bennett S. Kingsbury D. Jolley L. Brain T. (2000). Effects of postural constraints on children's catching behavior. Res. Q. Exerc. Sport 71 (1), 69–73. 10.1080/02701367.2000.10608882 10763523

[B22] Davids K. Glazier P. Araújo D. Bartlett R. (2003). Movement systems as dynamical systems. Sports Med. 33 (4), 245–260. 10.2165/00007256-200333040-00001 12688825

[B23] Dicks M. Davids K. Button C. (2009). Representative task design for the study of perception and action in sport. Int. J. Sport Psychol. 40, 506–524.

[B24] Farooq M. A. Parkinson K. N. Adamson A. J. Pearce M. S. Reilly J. K. Hughes A. R. (2018). Timing of the decline in physical activity in childhood and adolescence: Gateshead Millennium Cohort Study. Br. J. Sports Med. 52 (15), 1002–1006. 10.1136/bjsports-2016-096933 28288966 PMC6204977

[B25] Flôres F. S. Rodrigues L. P. Cordovil R. (2021). Development and construct validation of a questionnaire for measuring affordances for motor behavior of schoolchildren. J. Mot. Learn. Dev. 9 (3), 496–511. 10.1123/jmld.2020-0055

[B26] Franks R. R. King D. Bodine W. Chisari E. Heller A. 4 J. F. (2022). AOASM position statement on esports, active video gaming, and the role of the sports medicine physician. Clin. J. Sport Med. 32 (3), e221–e229. 10.1097/jsm.0000000000001034 35470342 PMC9042337

[B27] French B. Sycamore N. J. McGlashan H. L. Blanchard C. C. V. Holmes N. P. (2018). Ceiling effects in the Movement Assessment Battery for Children-2 (MABC-2) suggest that non-parametric scoring methods are required. PLOS ONE 13 (6), e0198426. 10.1371/journal.pone.0198426 29856879 PMC5983505

[B28] Gallahue D. Ozmun J. (2006). Understanding motor development; infants, children, adolescents, adults. 6th ed. Boston, MA: McGraw-Hill.

[B29] Giblin S. Collins D. Button C. (2014). Physical literacy: Importance, assessment and future directions. Sports Med. 44 (9), 1177–1184. 10.1007/s40279-014-0205-7 24898813

[B31] Guess T. M. Razu S. Jahandar A. Skubic M. Huo Z. (2017). Comparison of 3D joint angles measured with the kinect 2.0 skeletal tracker versus a marker-based motion capture system. J. Appl. Biomechanics 33 (2), 176–181. 10.1123/jab.2016-0107 27918704

[B32] Hallal P. C. Andersen L. B. Bull F. C. Guthold R. Haskell W. Ekelund U. (2012). Global physical activity levels: Surveillance progress, pitfalls, and prospects. Lancet 380 (9838), 247–257. 10.1016/s0140-6736(12)60646-1 22818937

[B33] Hardy L. L. Barnett L. Espinel P. Okely A. D. (2013). Thirteen-year trends in child and adolescent fundamental movement skills: 1997-2010. Med. Sci. Sports Exerc. 45 (10), 1965–1970. 10.1249/mss.0b013e318295a9fc 24048319

[B34] Henderson S. E. Sugden D. A. Barnett A. L. (2007). Movement assessment battery for children – 2 examiner’s manual. London: Harcourt Assessment.

[B35] Hu L. T. Bentler P. M. (1999). Cutoff criteria for fit indexes in covariance structure analysis: Conventional criteria versus new alternatives. Struct. Equ. Model. A Multidiscip. J. 6 (1), 1–55. 10.1080/10705519909540118

[B36] Hulteen R. M. Johnson T. M. Ridgers N. D. Mellecker R. R. Barnett L. M. (2015). Children's movement skills when playing active video games. Percept. Mot. Ski. 121 (3), 767–790. 10.2466/25.10.pms.121c24x5 26654991

[B37] Jak S. Oort F. J. Dolan C. V. (2014). Measurement bias in multilevel data. Struct. Equ. Model. A Multidiscip. J. 21 (1), 31–39. 10.1080/10705511.2014.856694

[B38] Jöreskog K. G. Sörbom D. (1993). Lisrel 8: Structural equation modeling with the SIMPLIS command language. Lincolnwood, IL: Scientific Software International, Inc.

[B39] Kaiser H. F. Rice J. (1974). Little jiffy, mark IV. Educ. Psychol. Meas. 34 (1), 111–117. 10.1177/001316447403400115

[B40] Kane M. T. (2013). Validating the interpretations and uses of test scores. J. Educ. Meas. 50 (1), 1–73. 10.1111/jedm.12000

[B41] Kim K. H. (2005). The relation among fit indexes, power, and sample size in structural equation modeling. Struct. Equ. Model. 12 (3), 368–390. 10.1207/s15328007sem1203_2

[B42] Kiphard E. J. Schilling F. (2007). Körperkoordinationstest für kinder: Ktk. Weinheim: Beltz-Test. 5511840

[B43] LeBlanc A. G. Chaput J. P. McFarlane A. Colley R. C. Thivel D. Biddle S. J. (2013). Active video games and health indicators in children and youth: A systematic review. PloS one 8 (6), e65351. 10.1371/journal.pone.0065351 23799008 PMC3683002

[B45] Logan S. W. Kipling Webster E. Getchell N. Pfeiffer K. A. Robinson L. E. (2015). Relationship between fundamental motor skill competence and physical activity during childhood and adolescence: A systematic review. Kinesiol. Rev. 4 (4), 416–426. 10.1123/kr.2013-0012

[B46] Logan S. W. Robinson L. E. Getchell N. Webster E. K. Liang L.-Y. Golden D. (2014). Relationship between motor competence and physical activity: A systematic review. Res. Q. Exerc. Sport 85 (S1), A–14.

[B47] Logan S. W. Robinson L. E. Wilson A. E. Lucas W. A. (2012). Getting the fundamentals of movement: A meta-analysis of the effectiveness of motor skill interventions in children. Child Care, Health Dev. 38 (3), 305–315. 10.1111/j.1365-2214.2011.01307.x 21880055

[B49] Lorenz R. C. Gleich T. Gallinat J. Kühn S. (2015). Video game training and the reward system. Front. Hum. Neurosci. 9 (40), 40–49. 10.3389/fnhum.2015.00040 25698962 PMC4318496

[B50] Lubans D. R. Morgan P. J. Cliff D. P. Barnett L. M. Okely A. D. (2010). Fundamental movement skills in children and adolescents review of associated health benefits. Sports Med. 40 (12), 1019–1035. 10.2165/11536850-000000000-00000 21058749

[B51] Luz C. Rodrigues L. P. Almeida G. Cordovil R. (2016). Development and validation of a model of motor competence in children and adolescents. J. Sci. Med. Sport 19 (7), 568–572. 10.1016/j.jsams.2015.07.005 26205772

[B52] MacCallum R. C. Browne M. W. Sugawara H. M. (1996). Power analysis and determination of sample size for covariance structure modeling. Psychol. methods 1 (2), 130–149. 10.1037/1082-989x.1.2.130

[B53] Mally K. K. Battista R. A. Robertson M. A. (2011). Distance as a control parameter for place kicking. J. Hum. Sport Exerc. 6 (1), 122–134. 10.4100/jhse.2011.61.14

[B54] Marsh H. W. Balla J. R. McDonald R. P. (1988). Goodness-of-fit indexes in confirmatory factor analysis: The effect of sample size. Psychol. Bull. 103 (3), 391–410. 10.1037/0033-2909.103.3.391

[B55] McDonald R. P. (1999). Test theory: A unified treatment. New York: Psychology Press.

[B56] Messick S. (1989). Meaning and values in test validation: The science and Ethics of assessment. Educ. Res. 18 (2), 5–11. 10.3102/0013189x018002005

[B57] Microsoft (2023). Azure kinect DK. Available at: https://azure.microsoft.com/en-gb/products/kinect-dk/#content-card-list-oc3409 .

[B58] Microsoft (2022). Kinect for Windows SDK 2.0. Available at: https://learn.microsoft.com/en-us/windows/apps/design/devices/kinect-for-windows .

[B59] Miller J. Vine K. Larkin D. (2007). The relationship of process and product performance of the two-handed sidearm strike. Phys. Educ. Sport Pedagogy 12 (1), 61–76. 10.1080/17408980601060291

[B60] Morley D. Rudd J. Issartel J. Goodway J. O’Connor D. Foulkes J. (2021). Rationale and study protocol for the movement oriented games based assessment (mogba) cluster randomized controlled trial: A complex movement skill intervention for 8–12 year old children within ‘made to play. Plos one 16 (6), e0253747. 10.1371/journal.pone.0253747 34166447 PMC8224954

[B61] Napoli A. Glass S. Ward C. Tucker C. Obeid I. (2017). Performance analysis of a generalized motion capture system using Microsoft Kinect 2.0. Biomed. Signal Process. Control 38, 265–280. 10.1016/j.bspc.2017.06.006

[B62] Newell K. M. McDonald P. V. Kugler P. N. (1991). “The perceptual-motor workspace and the acquisition of skill,” in Tutorials in motor neuroscience (Dordrecht: Springer), 95–108.

[B63] Ng J. L. Button C. Collins D. Giblin S. Kennedy G. (2020). Assessing the internal reliability and construct validity of the general movement competence assessment for children. J. Mot. Learn. Dev. 8 (1), 87–106. 10.1123/jmld.2018-0047

[B64] Ng J. L. Button C. (2018). Reconsidering the fundamental movement skills construct: Implications for assessment. Mov. Sport Sci. 102, 19–29. 10.1051/sm/2018025

[B65] Nunnally J. Bernstein I. (1994). Psychometric theory. 3rd ed. New York, NY: MacGraw-Hill.

[B66] Page Z. E. Barrington S. Edwards J. Barnett L. M. (2017). Do active video games benefit the motor skill development of non-typically developing children and adolescents: A systematic review. J. Sci. Med. Sport 20 (12), 1087–1100. 10.1016/j.jsams.2017.05.001 28600111

[B68] Pinder R. A. Davids K. Renshaw I. Araújo D. (2011). Representative learning design and functionality of research and practice in sport. J. Sport Exerc. Psychol. 33 (1), 146–155. 10.1123/jsep.33.1.146 21451175

[B69] Roberton M. A. Konczak J. (2001). Predicting children's overarm throw ball velocities from their developmental levels in throwing. Res. Q. Exerc. Sport 72 (2), 91–103. 10.1080/02701367.2001.10608939 11393884

[B70] Rudd J. Butson M. Barnett L. Farrow D. Berry J. Borkoles E. (2016). A holistic measurement model of movement competency in children. J. Sports Sci. 34 (5), 477–485. 10.1080/02640414.2015.1061202 26119031

[B71] Rudd J. R. Barnett L. M. Butson M. L. Farrow D. Berry J. Polman R. C. (2015). Fundamental movement skills are more than run, throw and catch: The role of stability skills. PLoS One 10 (10), 01402244–e140315. 10.1371/journal.pone.0140224 PMC4607429 26468644

[B72] Rudd J. R. Crotti M. Fitton-Davies K. O’Callaghan L. Bardid F. Utesch T. (2020a). Skill acquisition methods fostering physical literacy in early-physical education (SAMPLE-PE): Rationale and study protocol for a cluster randomized controlled trial in 5–6-year-old children from deprived areas of North West England. Front. Psychol. 11, 1228. 10.3389/fpsyg.2020.01228 32625143 PMC7311787

[B73] Rudd J. R. Pesce C. Strafford B. W. Davids K. (2020b). Physical literacy-A journey of individual enrichment: An ecological dynamics rationale for enhancing performance and physical activity in all. Front. Psychol. 11, 1904. 10.3389/fpsyg.2020.01904 32849114 PMC7399225

[B74] Scheuer C. Herrmann C. Bund A. (2019). Motor tests for primary school aged children: A systematic review. J. Sports Sci. 37, 1097–1112. 10.1080/02640414.2018.1544535 30604655

[B75] Schöllhorn W. I. Nigg B. M. Stefanyshyn D. J. Liu W. (2002). Identification of individual walking patterns using time discrete and time continuous data sets. Gait Posture 15 (2), 180–186. 10.1016/s0966-6362(01)00193-x 11869912

[B76] Seifert L. Button C. Davids K. (2013). Key properties of expert movement systems in sport. Sports Med. 43 (3), 167–178. 10.1007/s40279-012-0011-z 23329604

[B77] Seo N. J. Fathi M. F. Hur P. Crocher V. (2016). Modifying Kinect placement to improve upper limb joint angle measurement accuracy. J. Hand Ther. 29 (4), 465–473. 10.1016/j.jht.2016.06.010 27769844 PMC6701865

[B78] Sireci S. G. (2007). On validity theory and test validation. Educ. Res. 36 (8), 477–481. 10.3102/0013189x07311609

[B79] Smits-Engelsman B. C. M. Jelsma L. D. Ferguson G. D. (2017). The effect of exergames on functional strength, anaerobic fitness, balance and agility in children with and without motor coordination difficulties living in low-income communities. Hum. Mov. Sci. 55, 327–337. 10.1016/j.humov.2016.07.006 27423302

[B80] Smits-Engelsman B. Verbecque E. Denysschen M. Coetzee D. (2022). Exploring cultural bias in two different motor competence test batteries when used in african children. Int. J. Environ. Res. Public Health 19 (11), 6788. 10.3390/ijerph19116788 35682371 PMC9180268

[B81] Steiger J. H. Lind J. C. (1980). “Statistically based tests for the number of common factors,” in Tthe annual meeting of the psychometric society (Iowa City, IA: Psychometrica). Paper presented at the.

[B82] Stodden D. F. Goodway J. D. Langendorfer S. J. Roberton M. A. Rudisill M. E. Garcia C. (2008). A developmental perspective on the role of motor skill competence in physical activity: An emergent relationship. Quest 60 (2), 290–306. 10.1080/00336297.2008.10483582

[B83] Stodden D. Langendorfer S. Roberton M. A. (2009). The association between motor skill competence and physical fitness in young adults. Res. Q. Exerc. Sport 80 (2), 223–229. 10.1080/02701367.2009.10599556 19650387

[B85] Tester G. Ackland T. R. Houghton L. (2014). A 30-year journey of monitoring fitness and skill outcomes in physical education: Lessons learned and a focus on the future. Adv. Phys. Educ. 4, 127–137. 10.4236/ape.2014.43017

[B86] Tucker L. R. Lewis C. (1973). A reliability coefficient for maximum likelihood factor analysis. Psychometrika 38 (1), 1–10. 10.1007/bf02291170

[B87] Tyler R. Foweather L. Mackintosh K. A. Stratton G. (2018). A dynamic assessment of children’s physical competence: The dragon challenge. Med. Sci. sports Exerc. 50 (12), 2474–2487. 10.1249/mss.0000000000001739 30067588 PMC6282672

[B88] Ulrich D. A. (2000). TGMD 2–Test of gross motor development examiner’s manual. Austin, TX: University of Michigan.

[B89] van Waelvelde H. Peersman W. Lenoir M. Engelsman B. C. S. Henderson S. E. (2008). The movement assessment battery for children: Similarities and differences between 4-and 5-year-old children from Flanders and the United States. Pediatr. Phys. Ther. 20 (1), 30–38. 10.1097/pep.0b013e31815ee2b2 18300931

[B90] Vella S. A. Aidman E. Teychenne M. Smith J. J. Swann C. Rosenbaum S. (2023). Optimising the effects of physical activity on mental health and wellbeing: A joint consensus statement from sports medicine Australia and the Australian psychological society. J. Sci. Med. Sport 26, 132–139. 10.1016/j.jsams.2023.01.001 36737260

[B91] Wheaton B. Muthen B. Alwin D. F. Summers G. F. (1977). Assessing reliability and stability in panel models. Sociol. Methodol. 8, 84–136. 10.2307/270754

[B92] Wolf E. J. Harrington K. M. Clark S. L. Miller M. W. (2013). Sample size requirements for structural equation models: An evaluation of power, bias, and solution propriety. Educ. Psychol. Meas. 73 (6), 913–934. 10.1177/0013164413495237 PMC4334479 25705052

[B93] Zumbo B. D. Chan E. K. (Editors) (2014). Validity and validation in social, behavioral, and health sciences (London: Springer), 54.

