# Supplementary material for: Construct validation of a general movement competence assessment utilising active video gaming technology
Source: Front Bioeng Biotechnol. 2023 Apr 18;11:1094469. doi: 10.3389/fbioe.2023.1094469 (PMC10151535; doi:10.3389/fbioe.2023.1094469)
Supplement: Supplementary file 1 [file Table1.pdf]

Supplementary table 1: Games of the GMCA, targeted movement attributes and descriptions of games.

GMCA games	*Targeted movement attributes*	*Stages*	*Description of stages*	*Allocated time - seconds (s)*
1. Balance<br><br>**Maximum distance between limbs are automatically configured based data collected by the motion sensor.*	• Postural control<br>• Unimanual coordination<br>• Bimanual coordination<br>• Bilateral coordination	Stage 1 – One leg	Balance on one leg hand (randomised for either left or right) and move the other foot into a target zone and keep them within the target zone for 3 seconds. Next target zone appears after a successfully held pose.	30s
		Stage 2* – One leg, one hand	Balance one leg (like in stage 1) with the added complexity of needing to also place one hand (randomised for left or right) into another target zone for the hands. The feet and hands need to be in the target zone before the 3 second counter starts. Next randomised target zones appear after holding the position for 3 seconds.	40s
		Stage 3* – One leg, two hands	Balance one leg and two hands. Like in stage 2 but with both hands needed to be placed within target zones. This increases the complexity of the task and demands on postural control.	50s
2. Precision<br><br>**Each map has a different pathway for tracking*	• Postural control<br>• Hand-eye coordination<br>• Unimanual coordination<br>• Bilateral coordination<br>• Bimanual coordination	Stage 1 – Precision unimanual (6 maps*)	Control an avatar (a car) unimanually (right or left hand) and lead it though a designated path. If the avatar is led off the path, it resets to the initial starting position on screen. Maps presented progress with increasing difficulty and complexity based on tracking directions (e.g., vertical, horizontal, diagonal, combinations)	360s (60s per map)
		Stage 2 – Precision symmetrical (8 maps*)	Both hands track two separate cars (left and right hand) on the map in a symmetrical pathway. If either car strays off the track, it resets to the start line. Cars on both sides have to end at the same time. If distance between cars are too far apart (implying movement is not symmetrically synchronous), both cars are reset to the starting position.	480s (60s per map)
		Stage 3 – Precision asymmetrical (6 maps*)	Lead two cars (on left and right) through two asymmetrical pathways. The same constraints as stage 2 are applied (i.e., when the cars are reset)	360s (60s per map)

3. Control<br><br>**Speed and bounce of the ball has been programmed to be sensitive to the amount of force used to control it. Hitting it hard speeds the ball up and vice-versa.*	• Reaction<br>• Force control<br>• Hand-eye coordination<br>• Bilateral coordination<br>• Object-manipulation	Stage 1	Catch a ball that drops from the top of the playing screen (randomised placement) and control it by keeping it bouncing between both hands. Then, manipulate the ball and direct it towards a target (i.e., balloon). When the ball hits the target, another target appears in another randomised location. When a ball is lost (e.g. bounces out of control, is not caught), a new ball drops again.	50s
4. Swiftness	• Locomotion<br>• Agility<br>• Spatial awareness<br>• Bimanual coordination<br>• Force control	Stage 1	Starting from the centre of the game area, move to randomised floor targets. Floor targets are visually represented on the playing screen. Only locomotion in forward, backward and lateral directions are required in this stage.	90s
		Stage 2	Same as above but with additional diagonal movements required (e.g. moving from bottom-left to top-right of the game area)	90s
5. Interception	• Spatial awareness<br>• Hand-eye coordination<br>• Bimanual coordination	Stage 1	Save the spaceships and intercept asteroids. Save spaceships (static objects) by hovering a hand over it for 2 seconds. Intercept asteroids (randomised dynamic objects) by touching them to make them disappear. Stage begins with static asteroids (for 15 seconds) then increases in difficulty with asteroids moving at random speeds.	60s
